# Thermal response of two sexually dimorphic *Calopteryx* (Odonata) over an ambient temperature range

**DOI:** 10.1002/ece3.6864

**Published:** 2020-10-22

**Authors:** Gretchen D. Schreiner, Lucie A. Duffy, Jonathan M. Brown

**Affiliations:** ^1^ Grinnell College Grinnell Iowa USA

**Keywords:** *Calopteryx*, damselfly, Odonata, pigmentation, sexual dimorphism, sympatry, thermal imagery, thermal response

## Abstract

1. Organisms may internally or behaviorally regulate their body temperatures or conform to the ambient air temperatures. Previous evidence is mixed on whether wing pigmentation influences thermoregulation in various odonates.
2. We investigated the thermal response of sympatric North American *Calopteryx aequabilis* and *Calopteryx maculata* with a thermal imaging study across a 25°C ambient temperature range.
3. We found that regressions of thorax temperature on ambient temperature standardized by species had similar slopes for male and female *C. maculata*, but females were consistently 1.5°C warmer than males. In contrast, the sexes of *C. aequabilis* differed in slope, with *C. aequabilis* females having a slope less than 1.0 and males having a slope greater than 1.0.
4. We found that regressions of thorax temperature on ambient temperature standardized by sex had similar slopes for males and females of both species, but *C. maculata* females were consistently 2.1°C warmer than *C. aequabilis* females.
5. Given that *C. aequabilis* is strongly sexually dimorphic in pigment, but *C. maculata* is not, our findings suggest that wing pigmentation may influence thermal response rate in sympatric populations of both species.

## Graphical abstract

We investigated the thermal response of sympatric North American *Calopteryx aequabilis* and *Calopteryx maculata* with a thermal imaging study across a 25°C ambient temperature range. We found that (a) regressions of thorax temperature on ambient temperature standardized by species had similar slopes for male and female *C. maculata* but different slopes for *C. aequabilis*, with a female slope less than 1.0 and a male slope greater than 1.0; and (b) regressions of thorax temperature on ambient temperature standardized by sex had similar slopes for males and for females of both species. Given that *C. aequabilis* is strongly sexually dimorphic in pigment, but *C. maculata* is not, our findings suggest that wing pigmentation may influence thermal response rate in sympatric populations of both species.

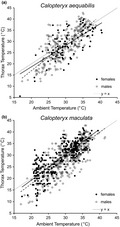

## 1 INTRODUCTION

Striking color variation within and between species is assumed to have visual signaling functions during mating interactions, either as a target of sexual selection or species recognition. However, the pigments and structural elements producing color variation may have additional, nonvisual consequences on fitness. For example, pigments have been hypothesized to act as antioxidants (Cooper, 2010; Cooper et al., 2016; McGraw, 2005), UV protection (Caldwell et al., 1998), or as nonfunctional excretion products from tryptophan breakdown (Linzen, 1974). Additionally, body color can affect solar energy absorption and so may influence body temperature in ectotherms (Ellers & Boggs, 2003; Lacey et al., 2010; Watt, 1968). These varied effects of color suggest that natural and sexual selection act on color polymorphic species.

Melanin is a common dark pigment in animals known to influence body temperature through absorption of solar radiation (Goulson, 1994; Gunn, 1998; Kingsolver & Wiernasz, 1991; Riley, 1997; Svensson & Waller, 2013; Watt, 1968, 1969). For example, dark wing color in insects has been hypothesized as a thermal adaptation associated with northern latitudes (Outomuro & Ocharan, 2011; Svensson & Waller, 2013; Watt, 1968, 1969) and high elevations (Loayza‐Muro et al., 2013) by increasing absorption of solar radiation. Sexually dimorphic melanized wing patterns may result in species‐specific thermal response curves across an ambient temperature range. These sexual dimorphisms in color increase proportionately with habitat difference between sexes, and their color variations can be predicted by canopy cover (a measure of UV radiation) (Cooper et al., 2016).

Pigment variation can thus interact with other means of thermoregulation in ectotherms, such as behavior. Closely related species or species with color polymorphisms provide an opportunity to evaluate the relative importance of coloration versus behavior and thermal consequences of other forms of selection on color. Although damselfly species can be highly sexually dimorphic in body and wing color, the evidence of a relationship between wing pigmentation and thermoregulation is mixed. *Calopteryx xanthostoma* male wing pigmentation does not influence temperature or solar radiation (Odonata: Calopterygidae) (Outomuro & Ocharan, 2011). *Enallagma cyathigerum* (Odonata: Zygoptera) males and females have similar heating and body temperature, and their female‐limited polymorphism is not maintained by temperature (Bots et al., 2008). Rubyspot *Hetaerina* damselfly wing spots do not influence thermoregulation (Odonata: Calopterygidae) (Rivas et al., 2016). However, northern damselfly populations tend to have more wing pigmentation, suggesting a thermoregulatory function (Outomuro & Ocharan, 2011). Finally, body temperatures of male *Pachydiplax longipennis* dragonflies increase with greater natural and artificial wing pigmentation (Moore et al., 2019).

Although wing pigmentation may be one predictor of body temperature, thermal response curves may also be impacted by a combination of other factors, including but not limited to body size, body color, intra‐ and interspecific behaviors, abiotic conditions, and environmental preferences (i.e., preferred perch location).

**Figure 1:**
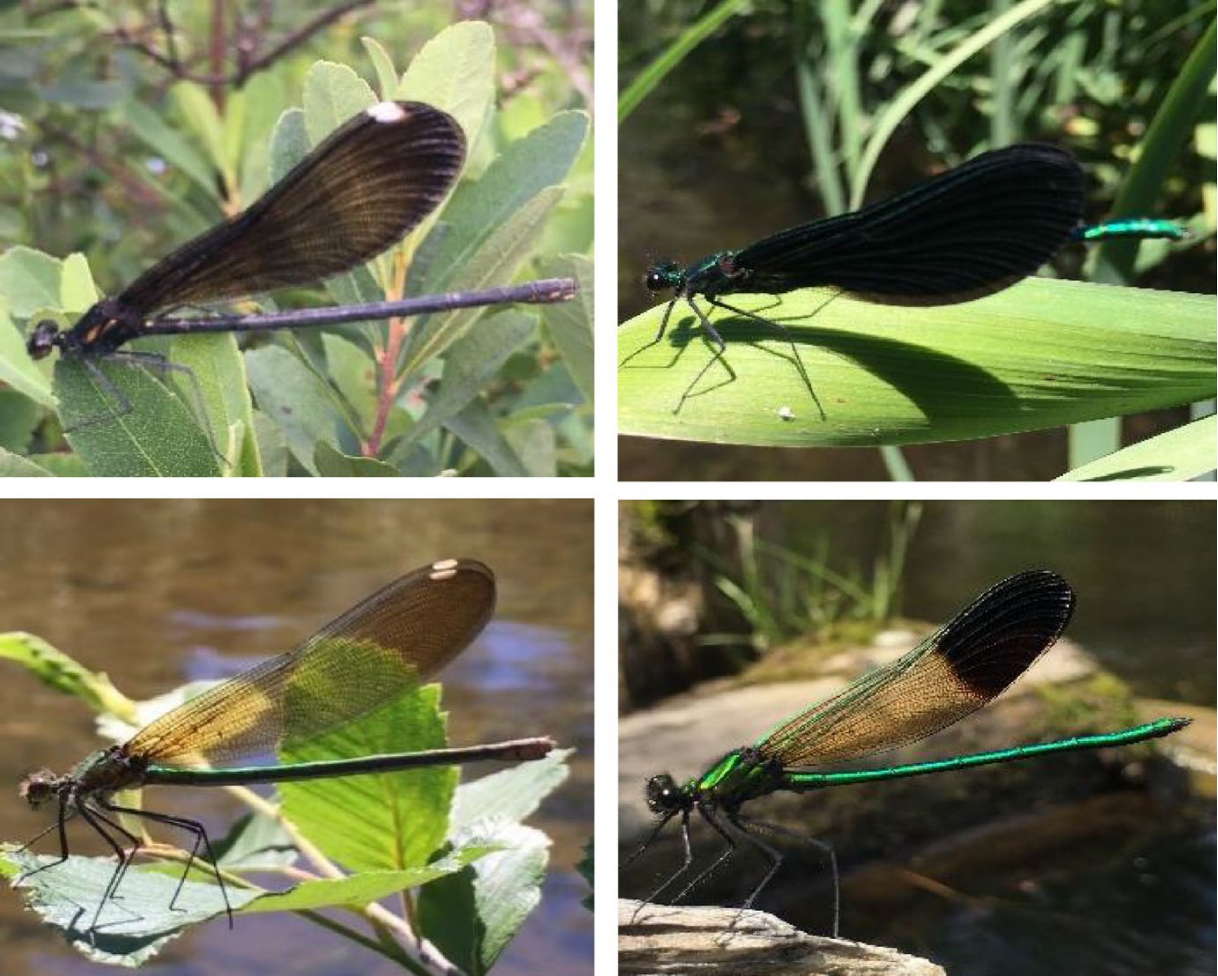
Sympatric *C. maculata* (female: top left, male: top right) and *C. aequabilis* (female: bottom left, male: bottom right) damselflies along the AuSable River near Grayling, MI, USA

We compared the thermal response of a related pair of North American sympatric Calopterygid damselflies with dimorphic wing pattern differences, *Calopteryx aequabilis* and *Calopteryx maculata*, using thermal imagery. Both sexes of *C. maculata* have completely pigmented wings. In contrast, *C. aequabilis* males have a dark spot only on the distal third of the wings, and these spots in females are much less pigmented. The body coloration of both species is similar, with males having iridescent green bodies, and females having dark brown or golden bodies (Figure 1). Both sexes and species are similarly sized, with *C. aequabilis* slightly larger than *C. maculata* and females' abdomens slightly larger than males' (Bland & Jaques, 2010).

We investigated whether the wing color and behavior differences between these two sympatric and closely related species influenced body temperature over an ambient temperature range experienced in the course of the day during mating season. For a given ambient temperature, one might expect that (a) *C. maculata*, the more melanized species, would have greater thorax temperatures than *C. aequabilis* and (b) males would have greater thorax temperatures than females, possibly due to greater sun exposure, wing melanization, and/or activity level.

## 2 MATERIALS AND METHODS

This study was conducted at three sites along the AuSable River near Grayling, MI, USA (Finley's Riverside Cabins [*N* = 589]: 44.657871, −84.728130; Burton's Landing [*N* = 66]: 44.663861, −84.647406; Keystone Landing [*N* = 538]: 44.665036, −84.627466) from 25 June 2018 to 2 July 2018 between 0800 and 1800 hr. At these sites, the river was slow‐moving and variable but mostly shallow in depth (0.5–1.5 m). Streams were 10–30 m wide, so damselflies were commonly exposed to full sun. These damselflies basked on vegetation along the banks and in the stream on rocks and debris dams, some of which had live vegetation. They roosted overnight in trees along the stream, and adult females oviposited in submerged vegetation. *C. aequabilis* was more common in faster flowing water (Burton's Landing and Keystone Landing), and *C. aequabilis* females were most scarce. Otherwise, all damselflies were extremely plentiful.

Surface temperature of perching damselflies was quantified from 20 to 30 cm distance with an infrared thermal camera (Seek Thermal Compact Pro) attached to the lightning port of an iPhone 6s (Apple). Thorax surface temperatures were measured using “Hi/Lo” mode (16:9 aspect ratio), as the thorax was usually the hottest point in the frame. Thorax temperatures of 89 *C. aequabilis* females, 294 *C. aequabilis* males, 324 *C. maculata* females, and 485 *C. maculata* males were measured. Only hardened adult individuals were measured (i.e., no tenerals). For a subset of individuals (*N* = 409), wing temperature was also recorded with manual “Spot” mode pointed at melanized regions. Damselflies were not marked, so individuals may have been measured more than once. However, the large population greatly decreased this chance of pseudoreplication.

Ambient air temperature was simultaneously measured within 1 m of each individual with a digital thermometer (Nielson‐Kellerman Kestrel 3000 Pocket Weather Meter).

This method is preferable to artificial temperature recording (i.e., using thermocouples) (I.A. Cooper et al., unpublished data), because it is more effective, less invasive, measures insects in their habitat, and predicts internal temperature with very high accuracy (Saastamoinen & Hanksi, 2008; Tsubaki et al., 2010), rendering it ideal for studies of small insects or populations with catch limits.

Analyses of covariance were performed to test for differences between species and sexes in regressions of thorax temperature on ambient temperature. We first tested a full model that included Ambient Temperature (covariate), Sex, Species, and all possible interaction terms. Because the three‐way interaction term was significant in the full model (Ambient Temperature * Species * Sex: *F*_1,1,185_ = 17.74, *p* < .0001), we then subset the full data by species and did separate analyses of sex differences within species. Finally, we subset the full data by sex and did separate analyses of species differences within sexes. Slopes for each species by sex combination were tested against an expectation of 1.0: a slope not different than 1.0 indicates a thermoconformer (Angilletta & Angilletta, 2009). Statistical analyses were performed in Minitab 18 (Minitab Inc, 2017) and SAS v.9.4 (SAS Institute Inc, 2016).

## 3 RESULTS

Wing temperatures were recorded for a subset of individuals; however, we chose to only analyze thorax temperatures because of the much larger sample size and strong Pearson correlation of 0.754 (95% CI [0.708, 0.793]) between wing and thorax temperature.

**Figure 2:**
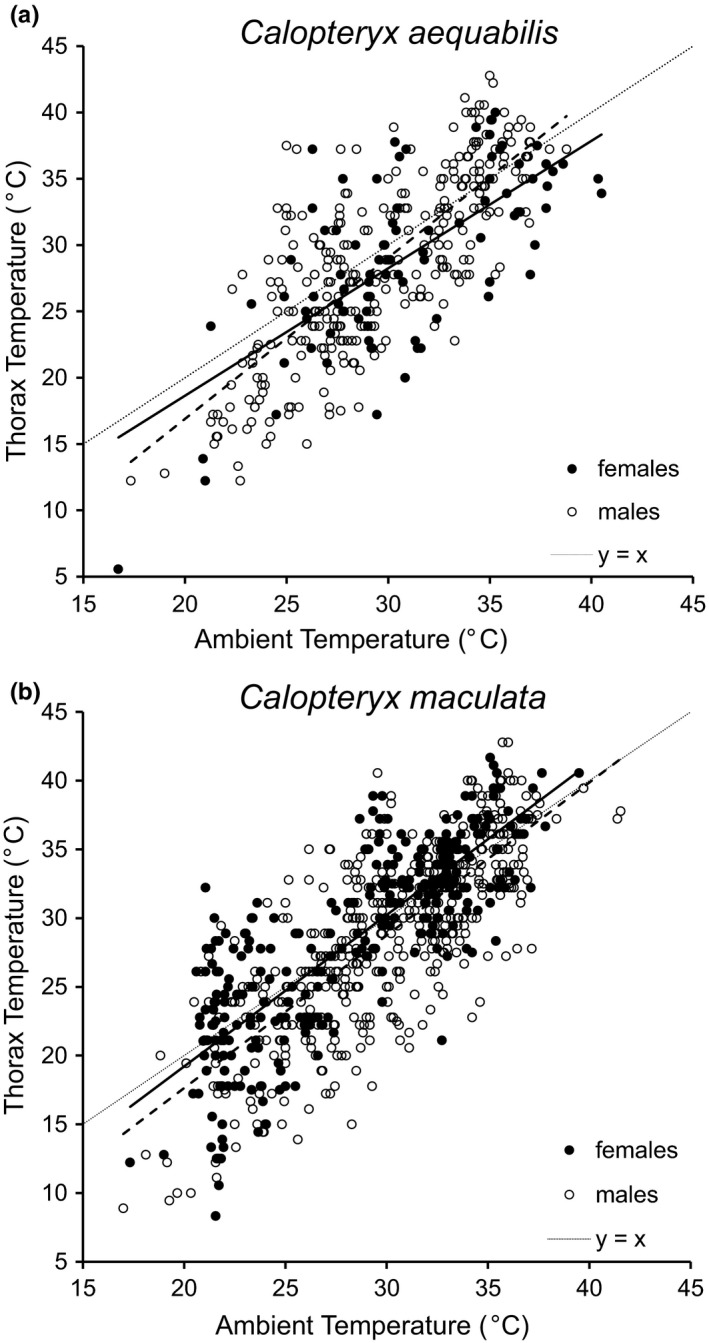
Effect of ambient air temperature on thorax surface temperature of (a) *C. aequabilis* and (b) *C. maculata* damselflies observed at the AuSable River in Michigan. Regression lines of female and male damselflies are shown with solid and dashed lines, respectively (Table 1). Fine dotted line represents perfect conformation to ambient temperature, a *y* = *x* line

Individual thorax temperatures deviated up to 11°C in both directions from ambient temperature. As expected, thorax temperature showed a strong and positive relationship with ambient air temperature for both sexes in both species (Ambient Temperature covariate in full model: *F*_1,1,185_ = 1,385.7, *p* < .0001; Figure 2). In the full model, at least one sex of one species had a significantly different regression slope than one other group of damselflies (Ambient Temperature * Species * Sex: *F*_1,1,185_ = 17.74, *p* < .0001).

Therefore, we subset the data by species and analyzed differences between the sexes standardized by species. The sexes of *C. aequabilis* displayed significantly different slopes (Figure 2a: Ambient Temperature * Sex: *F*_1,379_ = 4.34, *p* < .04). The slope for *C. aequabilis* females was not significantly different from 1.0, while the slope for male *C. aequabilis* was significantly greater than 1.0 (Table 1). Although thoraces of *C. aequabilis* males and females had similar temperatures at low ambient temperatures, male *C. aequabilis* thorax temperatures increased significantly faster than females' as ambient temperature increased. Consequently, *C. aequabilis* females were on average cooler than ambient temperature over most of the day (Figure 2a).

**Table 1:** Regression analyses of thorax temperature versus ambient temperature

Species	Sex	*df*	Slope (95% CI)	Intercept	*R* ^2^ (adj)
*C. aequabilis*	Female	88	0.961 (0.749, 1.173)	−0.58	0.476
	Male	293	1.207 (1.088,1.325)	−7.13	0.577
*C. maculata*	Female	324	1.102 (1.019, 1.186)	−2.83	0.676
	Male	485	1.112 (1.031, 1.193)	−4.61	0.599
	Both^a^	809	1.107 (1.049, 1.166)	−4.48	0.632
Both^b^	Female	412	1.075 (0.996, 1.154)	−2.06	0.637

^a^ excludes Ambient Temperature * Sex.

^b^ excludes Ambient Temperature * Species.

In contrast, the slopes for male and female *C. maculata* were not significantly different from one another (Figure 2b: Ambient Temperature * Sex: *F*_1,808_ = 0.03, *p* = .87). In addition, the 95% confidence intervals for the slopes of each sex did not include 1.0, indicating that both sexes had thorax temperatures that increased faster than ambient temperature (Table 1). In a model assuming equal slopes for the two sexes, the female regression line was also found to be significantly above that of males, with females' thoraces being on average 1.5°C warmer than males' for a given ambient temperature (Figure 2b, Table 1: Sex: *F*_1,808_ = 26.16, *p* < .0001).

We then analyzed differences between the species standardized by sex. The slopes for female *C. aequabilis* and *C. maculata* were not significantly different from one another (Ambient Temperature * Species: *F*_1,412_ = 1.91, *p* = .17). In a model assuming equal slopes for females of both species, the *C. maculata* regression line was found to be significantly above that of *C. aequabilis*, with *C. maculata* thoraces being on average 2.1°C warmer than *C. aequabilis* for a given ambient temperature (Table 1: Species: *F*_1,412_ = 17.16, *p* < .0001). Similarly, the slopes for male *C. aequabilis* and *C. maculata* were not significantly different from one another (Ambient Temperature * Species: *F*_1,778_ = 1.73, *p* = .19). In a model assuming equal slopes for males of both species, the regression line for *C. maculata* and *C. aequabilis* was not significantly different in elevation from one another (Species: *F*_1,778_ = 0.94, *p* = .33).

At 30°C, the optimal reproductive temperature of another damselfly (Tsubaki et al., 2010), thoraces of *C. aequabilis* males and females were 29.1°C (95% CI [25.5, 32.6]) and 28.5°C (95% CI [21.9, 34.6]), respectively, while *C. maculata* males and females had thorax temperatures of 28.7°C (95% CI [26.3, 31.2]) and 30.3°C (95% CI [27.7, 33.0]), respectively.

## 4 DISCUSSION

We investigated whether the color and behavior differences between *C. aequabilis* and *C. maculata* influenced thorax temperature over an ambient temperature range. For a given ambient temperature, one might expect that (a) *C. maculata*, the more melanized species, would have greater thorax temperatures than *C. aequabilis* and (b) males would have greater thorax temperatures than females, possibly due to greater sun exposure, wing melanization, and/or activity level. Supporting these expectations, we found that (a) *C. maculata* females were consistently warmer than the less melanized *C. aequabilis* females and (b) male *C. aequabilis* thorax temperatures increased at a greater rate than thoraces of the less melanized female *C. aequabilis*. Contrary to these expectations, our results indicated that (a) despite less saturated wing melanin, *C. maculata* females were consistently warmer than *C. maculata* males; (b) *C. aequabilis*, the species with less average wing melanization, was not consistently cooler than *C. maculata* across the ambient temperature range; and (c) despite greater wing melanization, *C. maculata* males had thermal response curves similar to the less melanized *C. aequabilis* males.

Body temperatures of both sexes in both species increased linearly with ambient air temperature, as expected of ectotherm thermoconformers that do not intensely thermoregulate (Angilletta & Angilletta, 2009). However, this first‐order relationship between ambient and thorax temperature does not describe all ectotherms, as some species behaviorally regulate to yield a curvilinear, second‐order relationship between these variables (Dreisig, 1984; Turner, 1987).

We typically imagine thermoregulation represented as a slope less than 1.0, where individuals maintain a constant internal temperature across an ambient temperature range. Surprisingly, we found that thorax temperatures of *C. maculata* males and females and *C. aequabilis* males increased at a rate greater than ambient. Based on past research with the *Mnais costalis* damselfly, 30°C is likely a reasonable reproductive thermal optimum for *C. aequabilis* and *C. maculata* (Tsubaki et al., 2010). Thus, these damselflies may boost their internal temperatures above ambient temperature during the reproductive period (the hottest part of the day) to achieve prime fitness, causing their slopes to increase. While the slope of the *C. aequabilis* females was less than 1.0, the intercept was greater than 0.0, which may indicate some form of thermoregulation, as well.

These strong relationships in *C. aequabilis* and *C. maculata* differed greatly from thermal relationships reported for a similar pair of European sympatric *Calopteryx* species (Svensson & Waller, 2013). In this European species pair, *Calopteryx splendens* and *Calopteryx virgo* have pigmentation that strikingly resembles *C. aequabilis* and *C. maculata*, respectively. In their study, they found significant slopes of 0.50 for *C. splendens* and −0.57 for *C. virgo*. However, these findings could be caused by a small sample size, a narrower ambient temperature range, and/or greater variation in environmental or behavioral factors than in our study. Interestingly, Svensson and Waller (2013) observed that *C. virgo* and *C. splendens* thoraces reached an upper asymptote of 35°C that suggested a negative consequence of overheating. However, we found no upper limit thorax temperatures for *C. maculata* or *C. aequabilis* at 35°C. This difference could result from dissimilar climates at field sites (Michigan, USA versus Sweden).

Despite the significant effect of ambient temperature, the span of residuals around our regression line was ~20°C. This large variation is expected of poikilotherms and can be explained by physiological and behavioral mechanisms. Physiological temperature management may occur by altering cellular melting points through phospholipid regulation (Farkas, 1984), exchanging heat between internal and surface‐level blood, and torpor (Hainsworth & Wolf, 1978). However, these species likely primarily thermoregulate with adaptive behavior or as a thermal consequence of activities important for fitness (i.e., displaying, entering water, and basking). Specifically, *C. aequabilis* males drop onto the water surface in courtship displays, and *C. aequabilis* females fully submerge while ovipositing (Conrad & Herman, 1987). *C. maculata* females primarily oviposit at the water's surface (Waage, 1984). We confirmed the occurrence of these behaviors in the field.

Additional field observations also supported the interpretation of behavioral thermoregulation for *C. maculata*. Explaining the similar regression slopes, male and female *C. maculata* had similar apparent activity levels and basking patterns. Namely, they appeared to bask equally on sunny debris dams in the stream and on shaded vegetation along the stream banks. Both sexes were less likely than *C. aequabilis* to return to a single territorial perch between flights. Since male and female *C. maculata* are likely to partially submerge in short periods during courtship displays and oviposition, respectively, it is possible that both sexes have similar exposure to any cooling effect of water. Male and female *C. maculata* wings are highly melanized, which may explain their similar heating rates in response to ambient temperatures. However, *C. maculata* females were 1.5°C hotter than males throughout the day. Since female *C. maculata* have larger abdomens than males, we hypothesize that this temperature difference is caused by a greater retention of heat as a consequence of their larger bodies.

Behavioral and phenotypic differences may also explain *C. aequabilis* thermal response curves. Our observations during the study indicated that *C. aequabilis* behavior differed by sex. Although both sexes claimed a specific perch, females spent more time basking, and males had greater activity with more time flying and defending territories, which could explain the steeper male slope. The tendency of *C. aequabilis* females to remain cooler than ambient temperature throughout the day could be explained by the following: (a) they are the only morph to completely submerge for long periods (oviposition); and (b) they are more likely to bask on the stream banks, where shaded cover is more common, than on the fully exposed debris dams in the streams' center. Finally, (c) the larger size of *C. aequabilis* compared with *C. maculata* could account for the heating rate and cooling ability of *C. aequabilis* males and females, respectively.

Since we only observed a significant sex effect on heating rate in *C. aequabilis*, the species with greater sexual dimorphism in pigment, and not *C. maculata*, the species with minimal sexual dimorphism in pigment, our findings suggest that wing pigmentation may influence thermal response rate in sympatric populations of both species. Namely, melanized wings add surface area to absorb heat, thereby creating boundary layers and changing the thermal microenvironment of the body as the damselfly perches.

Although wing pigmentation may drive thermal response, alternative explanations are possible. Each species' breeding system may differ, resulting in (a) distinct causes of wing pattern and (b) different types and locations of species‐specific consexual and heterosexual behaviors, altering how they respond to environmental conditions (Conrad & Herman, 1987; Waage, 1984). For instance, at midday, we observed that *C. aequabilis* males and females were less active than *C. maculata* and spent more time perching near the stream banks, while *C. maculata* males tended to more actively pursue mates in full sunlight. Additionally, sexually dimorphic body colors may also affect thorax temperature. For example, the brighter, iridescent bodies of males may absorb less heat through the cuticle than the darker, less reflective bodies of females. Thus, wing pigmentation may be a red herring; any other distinction between the species that may drive thermal response, like behavior, is likely correlated with the difference in wing pattern. These alternative hypotheses beg for future research on these variables to explain the mechanism of thermal response in *C. aequabilis* and *C. maculata*.

Additional study could explore the generalizability of these findings in the allopatric regions of these species or in another sympatric *Calopterygid* system. Marked individuals could be studied over time to generate specific time‐temperature curves. Temperature manipulation in controlled experiments with other damselfly species could determine if an upper temperature limit is specific to *C. virgo* and *C. splendens*. Field observation could quantitatively correlate behaviors to a thorax temperature versus ambient temperature curve. Quantifying the effect of other environmental factors, like UV index or wind speed, on thorax temperature could create a more complete regression model. Finally, studying mating frequency across an ambient temperature range could expose the sexual selection responsible for these thermoregulatory relationships, as other damselflies prefer specific internal temperatures in mates (Tsubaki et al., 2010). In addition to our study, these future directions can continue to elucidate biological consequences of pigment.

## CONFLICT OF INTEREST

None declared.

## AUTHOR CONTRIBUTIONS

**Gretchen Schreiner:** Conceptualization (equal); data curation (equal); formal analysis (lead); investigation (equal); methodology (equal); writing–original draft (lead); writing–review and editing (lead). **Lucie A. Duffy:** Conceptualization (equal); data curation (equal); investigation (equal); methodology (equal); writing–review and editing (supporting). **Jonathan Brown:** Conceptualization (equal); data curation (equal); formal analysis (supporting); funding acquisition (lead); methodology (equal); project administration (lead); resources (lead); supervision (lead); writing–review and editing (supporting).

## Data Availability

The data are available in the Dryad Data Repository (https://doi.org/10.5061/dryad.zpc866t72).

## References

[ece36864-bib-0001] Angilletta, M. J., Jr. , & Angilletta, M. J. (2009). Thermal adaptation: A theoretical and empirical analysis. Oxford University Press.

[ece36864-bib-0002] Bland, R. G. , & Jaques, H. E. (2010). How to know the insects (3rd ed.). Waveland Press.

[ece36864-bib-0003] Bots, J. , De Bruyn, L. , Van Damme, R. , & Van Gossum, H. (2008). Effects of phenotypic variation onto body temperature and flight activity in a polymorphic insect. Physiological Entymology, 33(2), 138–144. 10.1111/j.1365-3032.2008.00616.x

[ece36864-bib-0004] Caldwell, M. M. , Bjorn, L. O. , Bornman, J. F. , Flint, S. D. , Kulandaivelu, G. , Teramura, A. H. , & Tevini, M. (1998). Effects of increased solar ultraviolet radiation on terrestrial ecosystems. Journal of Photochemistry and Photobiology Biology, 46(1–3), 40–52. 10.1016/S1011-1344(98)00184-5

[ece36864-bib-0005] Conrad, K. F. , & Herman, T. B. (1987). Territorial and reproductive behavior of *Calopteryx aequabilis* say (Odonata: Calopterygidae) in Nova Scotia, Canada. Advances in Odonatology, 3, 41–50.

[ece36864-bib-0006] Cooper, I. A. (2010). Ecology of sexual dimorphism and clinal variation of coloration in a damselfly. The American Naturalist, 176(5), 566–572. 10.1086/656491 20860526

[ece36864-bib-0007] Cooper, I. A. , Brown, J. M. , & Getty, T. (2016). A role for ecology in the evolution of colour variation and sexual dimorphism in Hawaiian damselflies. Journal of Evolutionary Biology, 29(2), 418–427. 10.1111/jeb.12796 26575956

[ece36864-bib-0008] Dreisig, H. (1984). Control of body temperature in shuttling ectotherms. Journal of Thermal Biology, 9(4), 229–233. 10.1016/0306-4565(84)90001-9

[ece36864-bib-0009] Ellers, J. , & Boggs, C. L. (2003). The evolution of wing color:Male mate choice opposes adaptive wing colouration divergence in *Colias* butterflies. Evolution, 57(5), 1100–1106. 10.1111/j.0014-3280.2003.tb00319.x 12836826

[ece36864-bib-0010] Farkas, T. (1984). Adaptation of fatty acid composition to temperature: A study on carp (*Cyprinus carpio* L.) liver slices. Comparative Biochemistry and Physiology Part B: Comparative Biochemistry, 79, 531–535. 10.1016/0305-0491(84)90361-4 6518757

[ece36864-bib-0011] Goulson, D. (1994). Determination of larval melanization in the moth, *Mamestra brassicae*, and the role of melanin in thermoregulation. Heredity, 73(5), 471–479. 10.1038/hdy.1994.145

[ece36864-bib-0012] Gunn, A. (1998). The determination of larval phase coloration in the African armyworm, *Spodoptera exempta* and its consequences for thermoregulation and protection from UV light. Entomogologia Experimentalis Et Applicata, 86(2), 125–133. 10.1046/j.1570-7458.1998.00273.x

[ece36864-bib-0013] Hainsworth, F. R. , & Wolf, L. L. (1978). The economics of temperature regulation and torpor in nonmammalian organisms In Wang L. C. H., & Hudson J. W. (Eds.), Strategies in cold (pp. 147–184). Academic Press.

[ece36864-bib-0014] Kingsolver, J. G. , & Wiernasz, D. C. (1991). Seasonal polyphenism in wing‐melanin pattern and thermoregulatory adaptation in *Pieris* butterflies. The American Naturalist, 137(6), 816–830. 10.1086/285195

[ece36864-bib-0015] Lacey, E. P. , Lovin, M. E. , Richter, S. J. , & Herington, D. A. (2010). Floral reflectance, color, and thermoregulation: What really explains geographic variation in thermal acclimation ability of ectotherms? The American Naturalist, 175(3), 335–349. 10.1086/650442 20100107

[ece36864-bib-0016] Linzen, B. (1974). The tryptophan to ommochrome pathway in insects. Advances in Insect Physiology, 10, 117–246. 10.1016/S0065-2806(08)60130-7

[ece36864-bib-0017] Loayza‐Muro, R. A. , Marticorena‐Ruiz, J. K. , Palomino, E. J. , Merritt, C. , De Baat, M. L. , Gemert, M. V. , Verweij, R. A. , Kraak, M. H. S. , & Admiraal, W. (2013). Persistence of *Chironomids* in metal polluted Andean high altitude streams: Does melanin play a role? Environmental Science and Technology, 47(1), 601–607. 10.1021/es302779b 23190356

[ece36864-bib-0018] McGraw, K. J. (2005). The antioxidant function of many animal pigments: Are there consistent health benefits of sexually selected colourants? Animal Behaviour, 69, 757–764. 10.1016/j.anbehav.2004.06.022

[ece36864-bib-0019] Minitab Inc. (2017). Getting Started with Minitab 18. Minitab Inc.

[ece36864-bib-0020] Moore, M. P. , Lis, C. , Gherghel, I. , & Martin, R. A. (2019). Temperature shapes the costs, benefits and geographic diversification of sexual coloration in a damselfly. Ecology Letters, 22(3), 437–446. 30616297 10.1111/ele.13200

[ece36864-bib-0021] Outomuro, D. , & Ocharan, F. J. (2011). Wing pigmentation in *Calopteryx* damselflies: A role in thermoregulation? Biological Journal of the Linnean Society, 103(1), 36–44. 10.1111/j.1095-8312.2011.01641.x

[ece36864-bib-0022] Riley, P. A. (1997). Melanin. The International Journal of Biochemistry & Cell Biology, 29(11), 1235–1239. 10.1016/S1357-2725(9)00013-7 9451820

[ece36864-bib-0023] Rivas, M. , Martinez‐Meyer, E. , Munoz, J. , & Cordoba‐Aguilar, A. (2016). Body temperature regulation is associated with climatic and geographical variables but not wing pigmentation in two rubyspot damselflies (Odonata: *Calopterygidae*). Physiological Entymology, 41(2), 132–142. 10.1111/phen.12137

[ece36864-bib-0024] Saastamoinen, M. , & Hanksi, I. (2008). Genotypic and environmental effects on flight activity and oviposition in the Glanville fritillary butterfly. The American Naturalist, 171(6), 701–712. 10.1086/587531 18419339

[ece36864-bib-0025] SAS Institute Inc (2016). SAS:STAT User’s Guide. SAS Institute Inc.

[ece36864-bib-0026] Svensson, E. I. , & Waller, J. T. (2013). Ecology and sexual selection: Evolution of wing pigmentation in *Caeloptergid* damselflies in relation to latitude, sexual dimorphism, and speciation. The American Naturalist, 182(5), E174–E195. 10.1086/673206 24107378

[ece36864-bib-0027] Tsubaki, Y. , Samejima, Y. , & Siva‐Jothy, M. T. (2010). Damselfly females prefer hot males: Higher courtship success in males in sunspots. Behavioral Ecology and Sociobiology, 64(10), 1547–1554. 10.1007/s00265-010-0968-2

[ece36864-bib-0028] Turner, J. S. (1987). On the transient temperatures of ectotherms. Journal of Thermal Biology, 12(3), 207–214. 10.1016/0306-4565(87)90006-4

[ece36864-bib-0029] Waage, J. K. (1984). Female and male interactions during courtship in *Calopteryx maculata* and *C. dimidiate* (Odonata: Calopterygidae): Influence of oviposition behavior. Animal Behaviour, 32, 400–404. 10.1016/S0003-3472(84)80276-6

[ece36864-bib-0030] Watt, W. B. (1968). Adaptive significance of pigment polymorphisms in *Colias* Butterflies, I. Variation of melanin pigment in relation to thermoregulation. Evolution, 22(3), 437–458. 10.1111/j.1558-5646.1968.tb03985.x 28564757

[ece36864-bib-0031] Watt, W. B. (1969). Adaptive significance of pigment polymorphisms in *Colias* butterflies, II. Thermoregulation and photoperiodically controlled melanin variation in *Colias eurytheme* . Proceedings of the National Academy of Sciences, 63(3), 767–774. 10.1073/pnas.63.3.767 PMC223518 16591777

